# Digital Pathways to Reducing Depression Among Aging Populations Through the “Broadband China” Pilot Program: Quasi-Natural Experiment

**DOI:** 10.2196/79260

**Published:** 2025-10-27

**Authors:** Zhiying Li

**Affiliations:** 1 School of Government University of Chinese Academy of Social Sciences Beijing China

**Keywords:** digital infrastructure, depression, social networks, health inequality, middle-aged and older adults, difference-in-differences

## Abstract

**Background:**

With the rise of digital technology, infrastructure development has become vital for social welfare and public health. However, evidence on its effects on depressive symptoms among middle-aged and older adults remains limited.

**Objective:**

This study evaluates the impact of digital infrastructure development on depressive symptoms among middle-aged and older adults, focusing on underlying mechanisms, heterogeneous effects, and health inequalities.

**Methods:**

We use longitudinal data from the China Health and Retirement Longitudinal Study (CHARLS), 2011-2020 (N=56,211). Exploiting the quasi-natural experiment of the “Broadband China” pilot policy, we apply a difference-in-differences approach to estimate the effect on depressive symptoms. Mediation analysis follows the Baron-Kenny 3-step procedure, with bootstrap tests (95% CI) for robustness, and causal interpretation relies on standard assumptions for observational data. Subgroup analyses explore heterogeneity across age, education, and sex groups.

**Results:**

Our findings indicate that the “Broadband China” pilot significantly reduces depressive symptoms among middle-aged and older adults (−0.33, *P*<.01). The positive effect is primarily mediated through strengthened social networks, including increased family connection, close social interactions, and greater social participation. Heterogeneity analysis shows that the benefits for depression reduction are more pronounced among women (−0.38, *P*<.01), middle-aged adults (−0.41, *P*<.01), and those with lower levels of education (−0.33, *P*<.01). Moreover, the results suggest that digital infrastructure plays a compensatory role in mitigating health disparities, thereby reducing inequalities in depression outcomes (−0.01, *P*<.01).

**Conclusions:**

Digital infrastructure reduces depressive symptoms among aging populations mainly by strengthening social networks. Embedding infrastructure into long-term strategies, enhancing digital literacy, and integrating digital health services are key to promoting healthy aging and reducing inequalities.

## Introduction

### Background

Population aging has become a significant challenge to global socioeconomic development [1]. According to the United Nations’ 2024 World Population Prospects, by the end of the 21st century, the global population aged 65 years and older is expected to reach 2.2 billion, surpassing the population of children younger than 18 years, and this proportion will continue to rise. China’s aging process is particularly rapid, with the population aged 65 years and older accounting for 15.6% of the total in 2024, and this share is expected to accelerate in the coming decades. In this context, the health—particularly the mental health dimension, such as depression—of middle-aged and older adults is not only crucial for individual well-being but also profoundly impacts health care resource allocation, the stability of social security systems, and sustainable economic development.

In recent years, improvements in social security systems and advancements in medical technologies have somewhat enhanced the physical health of middle-aged and older adults, but depression has still not received adequate attention [2]. Depression is one of the most common psychological disorders among older adults. Studies show that the global prevalence of major depression is as high as 9.7% [3], while the depression rate for middle-aged and older adults in China is 24.1% [4], with severe depression accounting for 2.3% [5]. Depressive symptoms not only increase individual medical expenditures and social health care burdens [6] but may also impair the social functioning of middle-aged and older adults, reduce their quality of life, and further affect social welfare systems and macroeconomic development [7]. However, current health evaluation systems often underestimate the long-term socioeconomic impacts of depression, leading to its neglect in public policy and resource allocation [8]. In this context, how to effectively reduce depressive symptoms among middle-aged and older adults has become a key issue for advancing healthy aging.

Social networks are considered one of the most important factors affecting the depression status of middle-aged and older adults. As retirement, children’s migration for work, and the increased mobility of social networks take place, interactions with the outside world may decrease, leading to an increased sense of social isolation, which exacerbates depressive symptoms [9]. Social networks not only include direct face-to-face interactions but also extend to remote social connections facilitated by digital technology [10]. Existing studies show that middle-aged and older adults with lower levels of social networks are more prone to anxiety and depression, while actively maintaining social relationships helps reduce depressive symptoms [11]. The promotion of digital infrastructure development and the widespread availability of the internet have provided middle-aged and older adults with new social channels, alleviating social isolation to some extent. The application of digital technology can help reduce depression by enhancing social networks and increasing social support [12]. On the one hand, the internet allows middle-aged and older adults to maintain closer contact with their children and friends, reducing social alienation and thus lowering feelings of loneliness and risk of depression [13]. On the other hand, the use of social media and web-based interaction platforms expands their social networks, enhancing their sense of social participation and helping to alleviate depressive symptoms [14].

However, there is still controversy regarding the impact of digital technology on depression among middle-aged and older adults. One view holds that digital technology can compensate for the social interaction and participation deficits of middle-aged and older adults, thus reducing depressive symptoms [15]. Another view, based on the “substitution effect of presence,” argues that excessive reliance on digital technology may reduce real-world social interactions, weakening the emotional support provided by face-to-face communication, and thus negatively affecting depression outcomes [16]. Moreover, excessive use of social media may also lead to issues such as information overload and internet fraud, increasing anxiety and psychological stress [17]. Although these studies provide valuable insights into the relationship between digital technology and depression, there is still no consensus in the academic community regarding how digital infrastructure impacts the depressive symptoms of middle-aged and older adults. Current research mainly focuses on internet use at the individual level, with less attention paid to the systemic impact of digital infrastructure development as an exogenous variable. Additionally, the role of social networks in this process remains underexplored. Therefore, examining how digital infrastructure development influences the depressive symptoms of middle-aged and older adults through the enhancement of social networks is not only of significant academic value but also has practical implications for policy-making on healthy aging.

Based on this, this paper uses the quasi-natural experiment of the “Broadband China” pilot policy to systematically examine the effect of digital infrastructure development on depressive symptoms among middle-aged and older adults and explore the mechanisms through social networks. Compared with existing studies, the main contributions of this paper are as follows: First, rigorous identification leveraging policy shocks. Existing research has focused on the correlation between individual internet use and depression, making it difficult to overcome endogeneity issues. This paper uses the “Broadband China” pilot, applying the difference-in-differences (DID) method to estimate the effect of digital infrastructure development on depressive symptoms, providing more externally valid empirical evidence for policy optimization. Second, it reveals the role of social networks. Although social networks are considered an important factor influencing depression, few studies have focused on how digital infrastructure reshapes the social patterns of middle-aged and older adults and their implications for depressive symptoms. This paper focuses on how digital infrastructure enhances family connection, close social interactions, and social participation, improving social interaction levels, thereby alleviating loneliness and reducing depressive symptoms, filling a gap in the literature. Third, expanding the perspective of health inequality. This paper further examines the heterogeneous effects of digital infrastructure development across different sexes, ages, and socioeconomic groups and, by combining the Kakwani relative deprivation index, reveals how the digital divide affects inequality in depression outcomes among middle-aged and older adults. This analysis not only deepens the understanding of the social effects of digital infrastructure development but also provides empirical support for the government to develop targeted healthy aging policies.

### Policy Background and Theoretical Framework

#### Policy Background

As an essential component of digital infrastructure, broadband networks are not only the cornerstone of information technology development but also have a profound impact on residents’ psychological well-being and risk of depression. To accelerate the spread of broadband networks, the State Council released the “Broadband China Strategy and Implementation Plan” in August 2013, which emphasized promoting the nationwide construction of high-speed, secure, and green digital infrastructure through regional pilot projects. From 2014 to 2016, pilot cities were selected in 3 batches, gradually expanding the scope of broadband access services [18]. The core objective of this strategy was to improve network coverage and transmission speed to meet the demands of socioeconomic entities for high-quality internet services, while also promoting the integration of broadband networks with other infrastructure and public services through increased fiscal investment, government guidance, interdepartmental collaboration, and technological innovation [19].

In the field of depression and psychological well-being, the popularization of broadband networks has provided essential support for the social connections and emotional health of middle-aged and older adults. High-quality internet access not only enables them to maintain closer contact with family and friends, reducing feelings of social isolation, but also lays the foundation for the widespread use of web-based social platforms, community interaction, and remote psychological services. Through the application of digital technologies, older adults can more easily participate in community activities, social organizations, and volunteer programs, enhancing their sense of social participation and belonging, which in turn helps reduce depressive symptoms [20]. Therefore, the “Broadband China” strategy is not only an important initiative in advancing the nation’s informatization construction but also provides a natural quasi-experiment for researching how digital infrastructure influences depression among middle-aged and older adults.

#### Theoretical Framework

The theory of resocialization suggests that individuals must continually adapt to new social environments, roles, and norms throughout their lives to maintain physical and psychological health, particularly in preventing depression, and realize self-worth. However, traditional social structure changes, adjustments in intergenerational relationships, and role transitions after retirement present challenges for middle-aged and older adults, such as shrinking social networks and reduced social participation, which may exacerbate feelings of loneliness and psychological imbalance [21]. In this context, digital infrastructure construction, as an essential support for social modernization, provides middle-aged and older adults with new pathways for socialization [22], enabling them to access information more conveniently and expand their social interactions, thus reducing depression risk [23]. Existing studies have shown that internet use can effectively alleviate social isolation among middle-aged and older adults and improve their social adaptability and health [24]. By optimizing information accessibility and the convenience of social interactions, digital infrastructure offers middle-aged and older adults broader social channels, enhancing their adaptability to social changes. This not only reduces loneliness but also increases a sense of belonging and self-efficacy, thereby lowering depressive symptoms [25].

The theory of social support further elucidates the mechanism through which digital infrastructure construction affects depression outcomes of middle-aged and older adults. Social support includes emotional support, belonging, being valued, practical help, as well as information and guidance, all of which are vital external resources affecting mental health [26]. The improvement of digital infrastructure has facilitated efficient interactions among family members, allowing middle-aged and older adults to communicate more smoothly with children and friends, thus alleviating emotional loneliness due to generational gaps or physical separation [27]. The development of digital services, such as smart payment, web-based government services, and telemedicine, has reduced the uncertainty in daily life, enabling middle-aged and older adults to gain greater autonomy and security in social activities, health care, and financial transactions, helping individuals better understand and adjust their psychological states and enhancing their ability to cope with psychological stress. Based on this, the following hypothesis is proposed:

- **H1:** Digital infrastructure development reduces depressive symptoms among middle-aged and older adults.

Social networks refer to stable relational systems formed during individuals’ social interactions [28], typically consisting of both strong and weak ties. Strong ties are primarily found among close family members and intimate friends, characterized by strong emotional connections and stability [29], while weak ties include neighbors, community members, or interest groups with lower interaction frequencies. Although emotional bonds are weaker, weak ties offer greater information heterogeneity and facilitate resource acquisition and social integration [30]. An individual’s social network is not only a platform for exchanging information and resources but also a significant source of emotional support and social recognition. However, as people age, the social networks of middle-aged and older adults gradually shrink, leading to fewer social opportunities and increased loneliness, which negatively affects their depression outcomes [31]. Research indicates that emotional support has a significant impact on depressive symptoms among older adults [32], and the widespread adoption of digital infrastructure provides middle-aged and older adults with low-cost and convenient social channels, allowing them to reconstruct social connections through digital platforms such as social media and web-based communities, expanding their social circles and maintaining interactions within their existing social networks. On the one hand, this strengthens the strong-tie network between middle-aged and older adults and their families and friends, enabling them to maintain more frequent emotional connections despite geographical and temporal limitations [13]. On the other hand, it expands weak-tie networks, allowing them to connect with like-minded peers through web-based communities and interest forums, enhancing social interaction and social support [33]. This dual optimization mechanism not only improves emotional support but also increases social participation, effectively alleviating loneliness and psychological stress, thereby reducing depressive symptoms. Based on this, the following hypotheses are proposed:

- **H2:** Digital infrastructure development improves the depression outcomes of middle-aged and older adults by enhancing their social networks.
- **H2a:** Digital infrastructure development alleviates depressive symptoms among middle-aged and older adults by strengthening their family connections.
- **H2b:** Digital infrastructure development alleviates depressive symptoms among middle-aged and older adults by enhancing their close social interactions.
- **H2c:** Digital infrastructure development alleviates depressive symptoms among middle-aged and older adults by promoting their social participation.

As digital technologies continue to develop, the digital divide has become an important manifestation of social inequality, exacerbating economic inequality [34], knowledge inequality [35], and educational inequality [36], as well as having a profound impact on depression among different groups. Particularly in rural areas with underdeveloped digital infrastructure and among middle-aged and older adults with low incomes and educational levels, inadequate access to information often prevents them from obtaining necessary resources for depression prevention and intervention in a timely manner. Numerous studies have shown that information asymmetry is a key factor in the deterioration of depressive symptoms [37], especially regarding knowledge about depression, intervention measures, and access to medical resources [38]. The construction of digital infrastructure, by providing more convenient internet access and information channels, can help these disadvantaged groups break through information barriers and gain timely knowledge and web-based support related to depression, thus effectively narrowing the inequalities in depression outcomes caused by information asymmetry and regional differences.

The construction of digital infrastructure is not only a tool for the flow of information but also provides new avenues for the accumulation of social capital. Social capital theory posits that an individual’s depression status is closely related to the breadth and depth of their social support network [39]. For middle-aged and older adults, a lack of social support and feelings of isolation are major factors contributing to depressive symptoms, especially for economically disadvantaged groups. Traditional social support networks, constrained by geographical, cultural, and generational differences, often fail to meet the needs of these groups. The widespread use of digital technologies, particularly the internet and social media, offers middle-aged and older adults more social platforms and interaction channels, enabling them to break the limitations of traditional social networks and establish digital connections with other groups, gaining more emotional support and psychological guidance [40]. Through these digital platforms, older adults can form new social support networks, significantly reducing the inequalities in depression caused by differences in social capital. Based on this, the following hypothesis is proposed:

- **H3:** Digital infrastructure development helps reduce inequalities in depressive symptoms among middle-aged and older adults.

**Figure 1:**
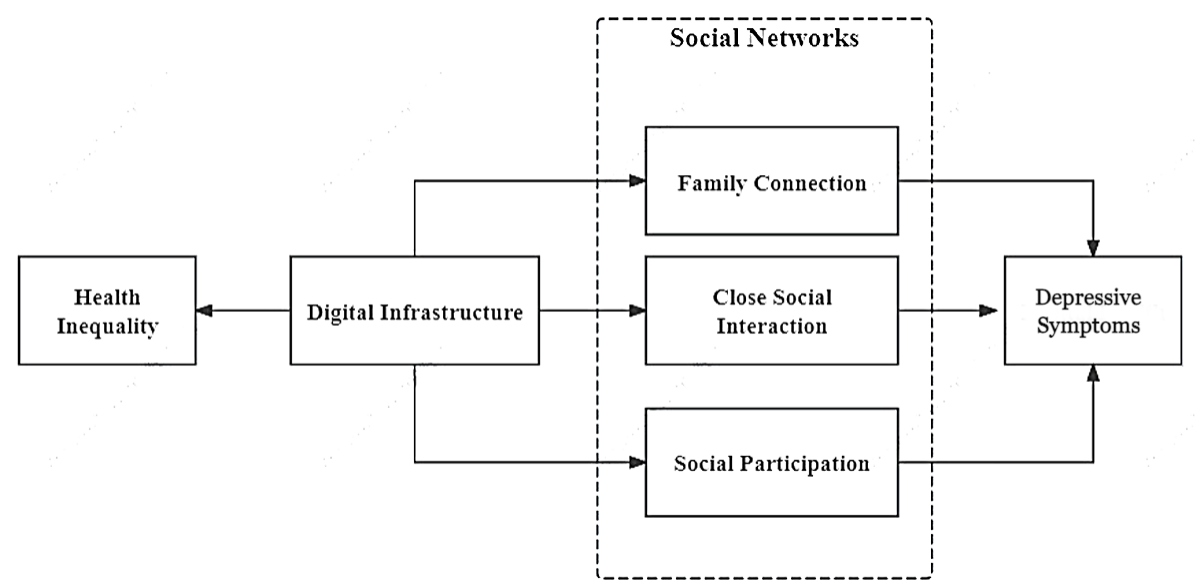
Research framework. This figure presents the conceptual framework of the study. All variables are measured in each wave of the panel data (2011, 2013, 2015, 2018, and 2020).

In summary, the research framework for this study is illustrated in Figure 1.

## Methods

### Data Source

The data for this study are drawn from the China Health and Retirement Longitudinal Study (CHARLS), a nationally representative survey conducted by the National School of Development at Peking University. Since 2011, CHARLS has carried out follow-up surveys every 2 to 3 years, covering 150 districts and counties nationwide through a multi-stage, probability-proportional-to-size sampling design [41,42]. The survey repeatedly collects extensive information on respondents—including demographic characteristics, socioeconomic conditions, health status, and family structure—ensuring comparability across waves.

This study uses 5 survey waves (2011, 2013, 2015, 2018, and 2020). The dependent variable, depressive symptoms (measured by the Center for Epidemiologic Studies Depression Scale [CES-D] 10 scale), is consistently available across all waves. Time-varying individual-level covariates—such as age, sex, marital status, education, family size, pension status, insurance coverage, and chronic disease status—are also updated in each wave. The key explanatory variable, digital infrastructure development, is constructed by linking city-level “Broadband China” pilot policy data to respondents based on their place of residence in each survey year.

The original pooled dataset contained more than 60,000 person-wave observations. To handle missing data, we applied listwise deletion, excluding respondents with incomplete information on the dependent variable or any covariates. The final analytic sample comprises 56,211 person-wave observations of adults aged 45 years and older. Because the proportion of missing cases was modest and not systematically patterned, the resulting sample remains broadly representative, and the risk of bias due to missing data is minimal.

### Ethical Considerations

The CHARLS project was reviewed and approved by the Institutional Review Board of Peking University (IRB00001052-11015). All participants, including middle-aged and older adults, provided written informed consent prior to participation. This study used deidentified, publicly available data, and all procedures complied with the ethical standards of the Declaration of Helsinki. Participants’ privacy and confidentiality were maintained throughout the study. No further ethics approval was required for this secondary data analysis.

### Variable Definitions

The core explanatory variable of this study is digital infrastructure development. According to the “Broadband China” Strategy and Implementation Plan issued by the State Council in 2013, the core goals of the strategy include enhancing broadband network coverage, optimizing network service quality, and promoting the development of digital applications to improve regional digital infrastructure. The implementation of the “Broadband China” strategy not only represents the government’s systematic investment in digital infrastructure but also has created significant regional infrastructure disparities, providing a suitable policy experiment environment for this study. Therefore, following the approaches of Peng et al [43], He et al [44], and Jia and Li [45], this study uses the “Broadband China” pilot policy as a proxy for digital infrastructure development. If middle-aged and older adult individual i in city c was included in the “Broadband China” pilot program in year t, the binary variable did is assigned a value of 1; otherwise, it is assigned a value of 0. For individuals who changed residence during the observation period, the assignment of did was based on the policy status of their current city in each survey wave, thereby dynamically capturing the actual local policy environment experienced by each respondent. Since the proportion of cross-city movers in the sample was very small, the potential influence of such cases on the estimation results is negligible.

The dependent variable in this study is depressive symptoms among middle-aged and older adults. Based on the work of Ma et al [46], depressive symptoms were measured using the CES-D. This scale includes 10 questions related to core symptoms and common manifestations of depression, such as: “I get upset over little things,” “I have trouble concentrating,” “I feel downhearted,” “I feel that things are an effort,” “I am hopeful about the future,” “I feel frightened,” “I have trouble sleeping,” “I feel happy,” “I feel lonely,” and “I feel like I can’t continue my life.” CHARLS asks respondents about their feelings and behaviors during the previous week, with response options ranging from “Rarely or none of the time (<1 day)” to “Most of the time (5-7 days).” Each item is scored from 0 to 3, with 2 items, “I am hopeful about the future” and “I feel happy,” being reverse-coded to align with the other items. The total score for these 10 items is the CES-D score, which serves as the depression outcome variable in this study, with a range from 0 to 30. A higher score indicates worse depressive symptoms. The CES-D has been widely applied in studies of middle-aged and older adults, and previous research has demonstrated its good reliability and validity [47,48].

The mechanism variable in this study is the social network of middle-aged and older adults, which includes family connections, close social interactions, and social participation. According to social network theory, the network is not only characterized by the breadth of relationships but also by their intimacy [30]. Therefore, the 3 dimensions selected in this study reflect varying degrees of intimacy in social relationships. Family connection, as the most intimate dimension of social contact, is measured by the frequency of contact with children. This frequency is based on the response to the question “How often do you see your children when you don’t live with them?” The response scale covers multiple time frames, ranging from “almost every day,” “two to three times per week,” “once a week,” “once every two weeks,” “once a month,” “once every three months,” “once every six months,” and “once a year,” to “almost never.” Thus, the measure captures the regularity of parent-child contact across daily, weekly, monthly, and annual intervals. Values range from 1 to 9, where a higher score indicates less frequent contact and reflects weaker family connections. For middle-aged and older adults with multiple children, the mean frequency across all children is used. Close social interactions represent looser social relationships and are measured by the response to “Have you visited friends or socialized in the past month?” A response of “Yes” is assigned a value of 1, and “No” is assigned a value of 0. Close social interactions reflect the frequency of interaction with nonfamily members, which typically offers emotional support and psychological relief, while indicating interaction with a more distant social circle. Social participation reflects broader social connections and indicates the extent of involvement in public and external social arenas. Social participation is measured by responses to whether, in the past month, the individual participated in activities such as “playing mahjong, chess, cards, or attending community events,” “joining social or volunteer activities,” or “attending educational or training courses.” A response of “Yes” is scored as 1, otherwise 0. The social participation score is the sum of these 4 activities, ranging from 0 to 4, indicating the individual’s involvement in social interaction and public affairs.

This study also controls for other factors that may affect the depressive symptoms of middle-aged and older adults, including individual characteristics (such as residence, sex, age, education level, ethnicity, pension insurance, health insurance, and chronic diseases) and family characteristics (such as marital status and household size). Furthermore, the study controls for time fixed effects and city fixed effects. All data management and statistical analyses were performed using Stata (version 18.0; Stata Corp). Descriptive statistics for the relevant variables are shown in Table 1, with additional descriptive statistics by survey wave reported in Multimedia Appendix 1.

**Table 1:** Variable definitions and descriptive statistics.

Variable type and variable name		Variable definition and assignment	Mean (SD)	Min	Max
**Dependent variable**					
	Depressive symptoms	A continuous variable is constructed based on the CES-D^a^ (higher score indicates more severe depressive symptoms)	8.223 (6.221)	0	30
**Core explanatory variable**					
	Did	“Broadband China” pilot policy × year	0.208 (0.406)	0	1
**Control variables**					
	Residence	Respondent’s residence (Urban = 0, Rural = 1)	0.597 (0.491)	0	1
	Sex	Respondent’s sex (Female = 0, Male = 1)	0.483 (0.5)	0	1
	Age (years)	Respondent’s actual age at the time of the survey	61.41 (10.008)	46	120
	Education	Respondent’s education (Below Primary = 1, Primary = 2, Secondary = 3, High School and Above = 4)	2.018 (1.062)	1	4
	Ethnicity	Ethnicity of Respondents (Non-Han = 0, Han = 1)	0.924 (0.266)	0	1
	Pension	Respondent’s pension insurance status (No pension insurance = 0, Any pension insurance = 1)	0.59 (0.492)	0	1
	Health insurance	Respondent’s health insurance status (No health insurance = 0, Any health insurance = 1)	0.948 (0.222)	0	1
	Chronic disease	Whether the respondent has chronic diseases (No = 0, Yes = 1)	0.764 (0.425)	0	1
	Marital status	Respondent’s marital status (Not married = 0, Married = 1)	0.856 (0.351)	0	1
	Household size	Number of family members of the respondent	3.176 (1.639)	1	16

^a^CES-D: Center for Epidemiologic Studies Depression Scale.

### Model Specification

In economic and social research, the DID method is commonly used to assess policy effects. The fundamental approach involves treating the policy pilot as an exogenous “quasi-natural experiment.” The implementation of the Broadband China pilot policy may generate pre- and postimplementation differences within the pilot regions, as well as differences between pilot and nonpilot regions at the same point in time. By performing regression analysis on these 2 types of differences, the net effect of the policy on the depressive symptoms of middle-aged and older adults can be effectively identified. Therefore, this study treats the Broadband China policy as a quasi-natural experiment and uses the DID method to examine its impact. The baseline regression model is specified as follows:

Depression*_ict_*=*α*_0_+*β*_1_DID_ct_+*β*_2_Controls*_ict_*+ *γ*_c_+*δ*_t_+*ε_ict_* (1)

Where the subscripts *i*, *c*, and *t* represent individual, city, and time, respectively. Depression*_ict_* is the dependent variable, indicating the level of depressive symptoms of middle-aged and older adult individual i in city c at year t. DID_ct_ is the core explanatory variable, representing whether city c implemented the “Broadband China” pilot in year t. It is the interaction term between the city-level treatment group indicator and the pre and postpilot implementation dummy variables for the “Broadband China” initiative. Controls*_ict_* represents the individual and household-level control variables. *γ*_c_ and *δ*_t_ are the city fixed effects and time fixed effects, respectively. *ε_ict_* is the random error term.

The validity of the estimation in equation 1 relies on the parallel trend assumption, which asserts that, in the absence of policy intervention, the dependent variable trends of the treatment and control groups should exhibit similar patterns. To test this assumption and explore the dynamic effects of the policy, the study adopts an event study approach, following the methodology of Beck et al [49]. The model for the dynamic effect analysis is as follows:



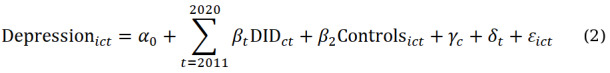



Where *β_t_* represents the estimated values corresponding to the years 2011-2020. The definitions of other variables are consistent with those in equation 1.

The Kakwani relative deprivation index [50] effectively quantifies disparities in depressive symptoms. According to the theory of relative deprivation, individuals with poorer health experience greater health deprivation and higher levels of health inequality. This study follows the methodologies of Li et al [41] and Turguttopbaş [51] and uses the Kakwani index to measure inequality in depressive symptoms. Specifically, since our data are panel in nature, the index was computed on a wave-by-wave basis (2011, 2013, 2015, 2018, and 2020). In each wave, the depressive symptom levels of middle-aged and older adults were ranked, and the relative deprivation value of each individual was calculated within that year’s reference group. As a result, every respondent has a time-varying relative deprivation value, reflecting their relative health position in each survey year. Formally, let Y represent the reference group in year t, with a sample size of n, and depressive symptom distribution vector *y*=(*y*_1_, *y*_2_, *y*_3_, *y*_n–1_, *y*_n_), where *y*_1_ ≤ *y*_2_ ≤ *y*_3_ ≤ *y*_n–1_ ≤ *y*_n_. Therefore, the relative deprivation index RD (*y*_j_, *y*_i_) of middle-aged and older adult individual i compared with individual j is defined as:



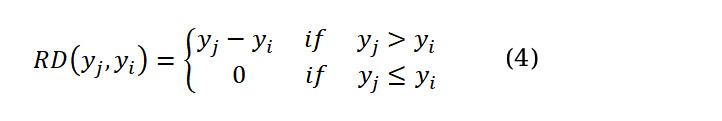



Based on equation 4, the average relative deprivation index of depressive symptoms for individual i in year t is calculated as follows:



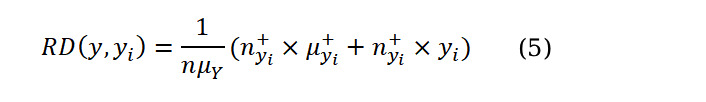



Where *μ*_y_ is the average depression score of all samples in the reference group Y, n_yi_^+^ is the number of middle-aged and older adults in the reference group Y whose health exceeds the health level of individual i (denoted as *y*_i_), and *μ*_yi_^+^ is the average health score of middle-aged and older adults in the reference group Y whose health exceeds *y_i_*.

To address potential multicollinearity, we calculated variance inflation factors (VIF) for all explanatory variables. All VIF values were well below the conventional threshold of 10 (mean VIF=1.17), suggesting that multicollinearity is not a concern. Model fit is evaluated using adjusted *R*^2^ (within), reported in each regression table.

## Results

### Baseline Regression

Table 2 presents the baseline regression results on the impact of digital infrastructure development on the depressive symptoms of middle-aged and older adults. In Model 1, which includes only the core explanatory variables, the coefficient of the interaction term is negative and statistically significant. Models 2 and 3 include individual and household characteristics, respectively, and the coefficient of the interaction term remains significant, indicating the robustness of the results. Model 4 further incorporates time and regional fixed effects. The coefficient of the interaction term is –0.3349 and is statistically significant at the 1% significance level. These results suggest that digital infrastructure development is associated with a significant reduction in depressive symptoms among middle-aged and older adults, thus supporting Hypothesis 1.

**Table 2:** Baseline regression results.

Variable			Model 1		Model 2		Model 3		Model 4
**Did**									
	β (95% CI)	–0.6073 (–0.7288 to –0.4858)		–0.4647 (–0.5824 to –0.3469)		–0.4799 (–0.5976 to –0.3622)		–0.3349 (–0.5055 to –0.1644)	
	*P* value	<.001		<.001		<.001		<.001	
**Sex**									
	β (95% CI)	—^a^		–1.7454 (–1.8462 to –1.6445)		–1.6422 (–1.7435 to –1.5410)		–1.7217 (–1.8661 to –1.5772)	
	*P* value	—		<.001		<.001		<.001	
**Residence**									
	β (95% CI)	—		1.2259 (1.1219 to 1.3299)		1.2385 (1.1347 to 1.3422)		0.8625 (0.6738 to 1.0513)	
	*P* value	—		<.001		<.001		<.001	
**Age (years)**									
	β (95% CI)	—		0.0219 (0.0154 to 0.0283)		0.0068 (0.0002 to 0.0135)		0.0130 (0.0045 to 0.0215)	
	*P* value	—		<.001		.05		.003	
**Education**									
	β (95% CI)	—		–0.7438 (–0.7942 to –0.6933)		–0.7327 (–0.7830 to –0.6823)		–0.6442 (–0.7184 to –0.5701)	
	*P* value	—		<.001		<.001		<.001	
**Ethnicity**									
	β (95% CI)	—		–0.4684 (–0.6942 to –0.2426)		–0.4512 (–0.6764 to –0.2261)		–0.2496 (–0.7019 to 0.2026)	
	*P* value	—		<.001		<.001		.28	
**Pension**									
	β (95% CI)	—		–0.2344 (–0.3517 to –0.1172)		–0.2155 (–0.3326 to –0.0984)		–0.3700 (–0.4959 to –0.2441)	
	*P* value	—		<.001		<.001		<.001	
**Health insurance**									
	β (95% CI)	—		–0.7111 (–0.9560 to –0.4661)		–0.6147 (–0.8592 to –0.3702)		–0.5423 (–0.8237 to –0.2609)	
	*P* value	—		<.001		<.001		<.001	
**Chronic disease**									
	β (95% CI)	—		2.2477 (2.1329 to 2.3624)		2.2626 (2.1481 to 2.3771)		2.0488 (1.9152 to 2.1824)	
	*P* value	—		<.001		<.001		<.001	
**Marital status**									
	β (95% CI)	—		—		–1.3494 (–1.5068 to –1.1921)		–1.2600 (–1.4946 to –1.0255)	
	*P* value	—		—		<.001		<.001	
**Household size**									
	β (95% CI)	—		—		–0.0339 (–0.0645 to –0.0032)		–0.0669 (–0.1041 to –0.0296)	
	*P* value	—		—		.03		<.001	
**_cons**									
	β (95% CI)	8.1897 (8.1325 to 8.2470)		7.9973 (7.4786 to 8.5160)		10.0024 (9.4298 to 10.5750)		9.7019 (8.8907 to 10.5132)	
	*P* value	<.001		<.001		<.001		<.001	
Time-fixed effects			No		No		No		Yes
Regional-fixed effects			No		No		No		Yes
N			56,211		56,211		56,211		56,211
Adjusted *R*^2^			0.0017		0.0952		0.1001		0.1421

^a^Not available.

### Robustness Check

#### Overview

To ensure the robustness of the research findings, multiple methods were used for robustness checks, including the parallel trend test, placebo test, propensity score matching difference-in-differences (PSM-DID), substitution of the dependent variable, and sample period adjustment.

#### Parallel Trend Test

**Figure 2:**
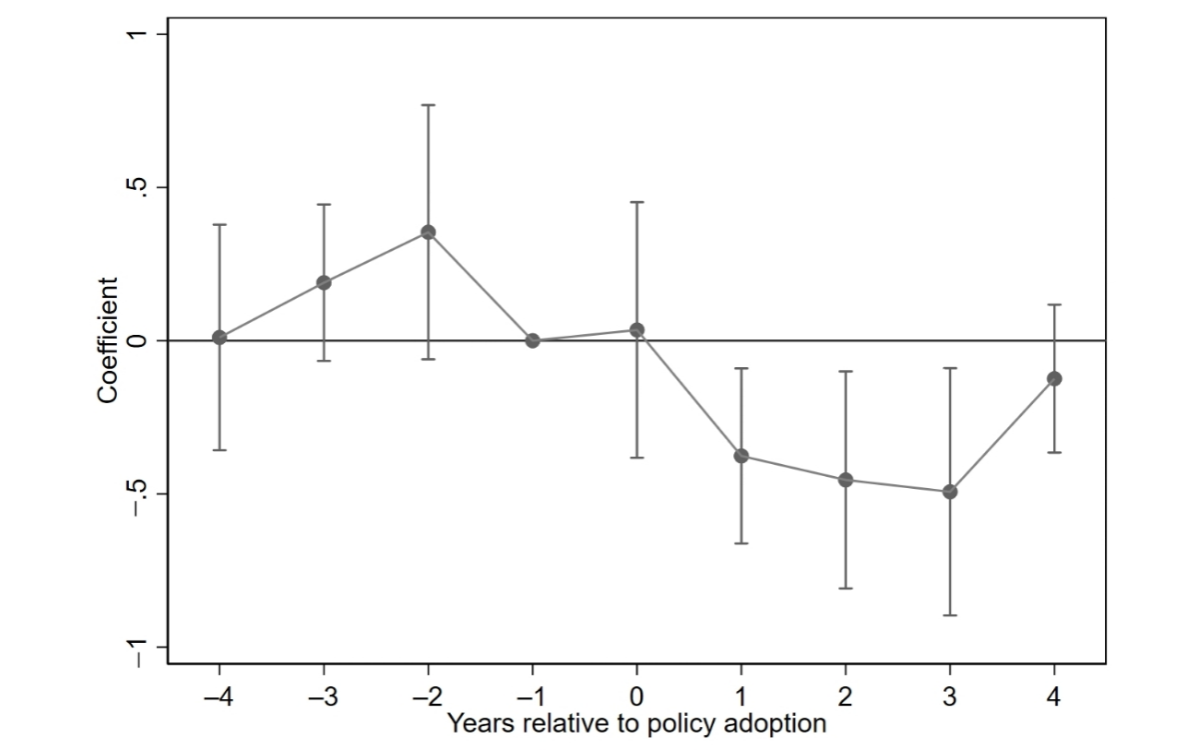
Parallel trend test.

The DID method relies on the parallel trend assumption, which requires that the depressive symptom trends of the treatment and control groups are similar prior to policy implementation. To examine this, we use an event study approach using the prepolicy period immediately before implementation (event=−1) as the reference. As a result, no coefficient or CI is reported for this baseline period. Figure 2 plots the estimated coefficients and their 95% CIs, showing that the prepolicy coefficients fluctuate around zero and are not statistically significant, suggesting no systematic differences in depressive symptom trajectories between the 2 groups.

The corresponding regression results are reported in Table 3. Furthermore, a joint *F* test of the prepolicy coefficients (event –4, event –3, and event –2) yields *F*_3, 16,981_ of 1.81 with a *P* value of .143, indicating that they are not jointly significant. Taken together, these results support the validity of the parallel trend assumption.

**Table 3:** Event study estimates for the parallel trends test.

Variables	Coefficient	SE	*T*	*P* value	95% CI
Event –4	0.010	0.188	0.06	.96	–0.358 to 0.379
Event –3	0.189	0.130	1.45	.15	–0.066 to 0.444
Event –2	0.354	0.212	1.67	.09	–0.061 to 0.769
Event –1	—^a^	—	—	—	—
Event 0	0.035	0.213	0.16	.87	–0.382 to 0.452
Event 1	–0.376	0.146	–2.58	.01	–0.662 to –0.090
Event 2	–0.455	0.181	–2.51	.01	–0.809 to –0.100
Event 3	–0.493	0.206	–2.40	.02	–0.897 to –0.090
Event 4	–0.124	0.123	–1.01	.31	–0.366 to 0.117
Control variables	Yes	Yes	Yes	Yes	Yes
_cons	9.744	0.428	22.77	<.001	8.905 to 10.583

^a^Omitted.

#### Placebo Test

**Figure 3:**
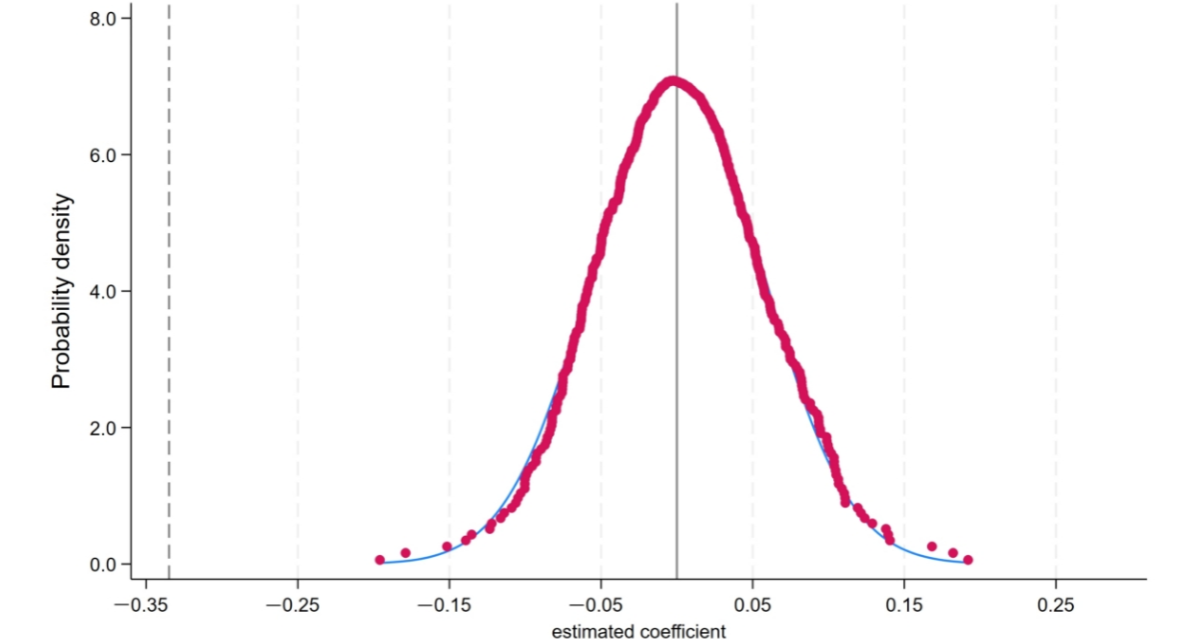
Placebo test.

To further verify the robustness of the policy effect and eliminate the interference of unobservable factors, this study randomly selected the experimental group and the policy pilot period to generate a simulated interaction term. A placebo test was conducted by randomly sampling the “Broadband China” policy interaction term 500 times. Figure 3 presents the kernel density distribution of the 500 random regression coefficients and the scatter plot of the *P* values. The results show that the mean of the simulated interaction term coefficients is close to zero, with a distribution that approximates normality. Most *P* values are greater than 0.1 and do not reach statistical significance. This indicates that the effect of the “Broadband China” policy is not influenced by sample selection bias, supporting the robustness of the baseline regression results.

#### PSM-DID

To control for selection bias between pilot and nonpilot regions, this study uses the PSM-DID method for robustness checks. Using nearest neighbor matching (1:4) and local linear regression matching, samples from nonpilot regions with similar socioeconomic characteristics are selected as the control group. DID regression is then implemented on the matched samples. The results from Models 1-2 in Table 4 show that the DID estimate coefficient for the Broadband China pilot policy remains significantly negative, further confirming the beneficial effect of digital infrastructure development on depressive symptoms among middle-aged and older adults.

**Table 4:** Robustness checks: propensity score matching difference-in-differences (PSM-DID), substitution of the dependent variable, and sample period adjustment.

Variable		Model 1 (PSM-DID)	Model 2 (PSM-DID)	Model 3 (substitution of the dependent variable)	Model 4 (sample period adjustment)
**Did**					
	β (95% CI)	–0.3713 (–0.5480 to –0.194)	–0.4219 (–0.6010 to –0.2429)	0.0266 (0.0028 to 0.0504)	–0.4312 (–0.6136 to –0.2488)
	*P* value	<.001	<.001	.03	<.001
Control variables		Yes	Yes	Yes	Yes
**_cons**					
	β (95% CI)	9.6373 (8.6974 to 10.5770)	9.7868 (8.6852 to 10.8885)	3.3162 (3.2140 to 3.4184)	9.9433 (9.0575 to 10.8290)
	*P* value	<.001	<.001	<.001	<.001
Time-fixed effects		Yes	Yes	Yes	Yes
Regional-fixed effects		Yes	Yes	Yes	Yes
*N*		50,852	48,048	55,405	42,933
Adjusted *R*^2^		0.1425	0.1428	0.0548	0.1358

#### Substitution of the Dependent Variable

Although life satisfaction and depression symptoms are conceptually distinct, prior studies have shown that life satisfaction is strongly correlated with mental health outcomes and is often treated as an important dimension of psychological well-being [52,53]. To verify the robustness of the results, this study substitutes life satisfaction indicators for the CES-D scale to measure the mental health status of middle-aged and older adults. The range of this indicator is from 1 to 5, with higher values indicating higher life satisfaction. The results from Model 3 in Table 4 show that after replacing the CES-D with life satisfaction, digital infrastructure development remains significantly associated with better psychological outcomes among middle-aged and older adults. While this substitution should be interpreted with caution, the consistency of results across different indicators enhances confidence in the robustness of our findings.

#### Sample Period Adjustment

The COVID-19 pandemic has had a profound impact on global public health systems and individual health conditions, potentially interfering with the identification of policy effects. To exclude this potential impact, this study excludes data from 2020 and reassesses the effect of digital infrastructure development on the depressive symptoms of middle-aged and older adults. The regression results from Model 4 in Table 4 show that after excluding the 2020 sample, the estimated coefficient of the “Broadband China” pilot policy is –0.4312, and it remains significant at the 1% level, further confirming the positive association between digital infrastructure development and reduced depressive symptoms among middle-aged and older adults.

### Mechanism Testing

To explore the mechanisms through which digital infrastructure development affects the depressive symptoms of middle-aged and older adults, this study introduces social network as a mediator, dividing it into 3 dimensions: family connection, close social interactions, and social participation. The regression results in Table 5 show that digital infrastructure development significantly promotes these 3 dimensions of the social network, thereby further reducing depressive symptoms among middle-aged and older adults, confirming the mediating effect.

To test the significance of the mediation effect, this study uses the Bootstrap method. If the CI of the indirect effect does not contain zero, it indicates that the effect is significant. Table 6 shows that the indirect effects of family connection (95% CI –0.0256 to –0.0038), close social interactions (95% CI –0.0162 to –0.0030), and social participation (95% CI –0.0290 to –0.0029) are all significant. These results are consistent with the previous regression analysis, indicating that digital infrastructure development effectively improves psychological well-being by mitigating depressive symptoms through strengthened social networks in various dimensions.

**Table 5:** Mechanism test.

Variable		Model 1 (family connection)	Model 2 (mental health)		Model 3 (close social interaction)		Model 4 (mental health)		Model 5 (social participation)		Model 6 (mental health)
**Did**											
	β (95% CI)	–0.1004 (–0.1718 to –0.0290)	–0.3644 (–0.5765 to –0.1523)	0.0235 (0.0078 to 0.0392)		–0.3259 (–0.4963 to –0.1554)		0.0209 (0.0052 to 0.0366)		–0.4158 (–0.5981 to –0.2335)	
	*P* value	.006	.001	.003		<.001		.009		<.001	
**Family connection**											
	β (95% CI)	—^a^	0.1466 (0.1120 to 0.1811)	—		—		—		—	
	*P* value	—	<.001	—		—		—		—	
**Close social interactions**											
	β (95% CI)	—	—	—		–0.4090 (–0.5179 to –0.3000)		—		—	
	*P* value	—	—	—		<.001		—		—	
**Social participation**											
	β (95% CI)	—	—	—		—		—		–0.7632 (–0.8843 to –0.6422)	
	*P* value	—	—	—		—		—		<.001	
Control variables		Yes	Yes		Yes		Yes		Yes		Yes
**_cons**											
	β (95% CI)	7.1214 (6.7989 to 7.4439)	8.6123 (7.6721 to 9.5525)	0.5679 (0.5102 to 0.6255)		9.9346 (9.1209 to 10.7484)		0.2741 (0.2023 to 0.3458)		10.1532 (9.2701 to 11.0363)	
	*P* value	<.001	<.001	<.001		<.001		<.001		<.001	
Time-fixed effects		Yes	Yes		Yes		Yes		Yes		Yes
Regional-fixed effects		Yes	Yes		Yes		Yes		Yes		Yes
*N*		42,364	42,364		56,209		56,209		42,931		42,931
Adjusted *R*^2^		0.1960	0.1390		0.0393		0.1431		0.0779		0.1392

^a^Not available.

**Table 6:** Mediation effect test based on the bootstrap method.

Mediator variable and effect type		Coefficient	SE	95% CI
**Family connections**				
	Indirect effect	–0.0147	0.0056	–0.0256 to –0.0038
	Direct effect	–0.3644	0.1237	–0.6069 to –0.1219
**Intimate social interaction**				
	Indirect effect	–0.0096	0.0034	–0.0162 to –0.0030
	Direct effect	–0.3259	0.0880	–0.4984 to –0.1534
**Social participation**				
	Indirect effect	–0.0159	0.0067	–0.0290 to –0.0029
	Direct effect	–0.4158	0.1048	–0.6212 to –0.2104

### Heterogeneity Analysis

Given the considerable differences in socioeconomic characteristics, health conditions, and levels of digital technology acceptance among middle-aged and older adults, the impact of digital infrastructure development on depressive symptoms is likely to vary across subgroups. Table 7 reports the heterogeneous effects. The results indicate that the policy significantly reduces depressive symptoms among the middle-aged (Model 1) and individuals with lower levels of education (Model 3), with coefficients of –0.4084 (*P*<.01) and –0.3300 (*P*<.01), respectively. In contrast, the effects for older adults (Model 2) and those with higher education (Model 4) are weaker and statistically insignificant, with coefficients of –0.2599 (*P*>.1) and –0.3769 (*P*>.05), suggesting that the policy’s benefits for depressive symptom reduction are not robust in these groups.

**Table 7:** Heterogeneity analysis.

Variable			Model 1 (age≤65 years)		Model 2 (age>65 years)		Model 3 (low education)		Model 4 (high education)		Model 5 (female)		Model 6 (male)
**Did**													
	β (95% CI)	–0.4084 (–0.6175 to –0.1993)		–0.2599 (–0.6059 to 0.0860)		–0.3300 (–0.5156 to –0.1443)		–0.3769 (–0.7894 to 0.0355)		–0.3794 (–0.6341 to –0.1247)		–0.2931 (–0.5189 to –0.0673)	
	*P* value	<.001		.14		<.001		.07		.004		.01	
Control variables			Yes		Yes		Yes		Yes		Yes		Yes
**_cons**													
	β (95% CI)	8.6117 (7.5430 to 9.6805)		11.5659 (9.7401 to 13.3917)		9.8586 (8.9596 to 10.7576)		6.6861 (4.8701 to 8.5021)		10.4365 (9.2335 to 11.6395)		7.4745 (6.3568 to 8.5922)	
	*P* value	<.001		<.001		<.001		<.001		<.001		<.001	
Time-fixed effects			Yes		Yes		Yes		Yes		Yes		Yes
Regional-fixed effects			Yes		Yes		Yes		Yes		Yes		Yes
*N*			38,331		17,880		49,216		6995		28,436		27,775
Adjusted *R*^2^			0.1501		0.1355		0.1334		0.0937		0.1304		0.1028

Turning to sex, digital infrastructure development significantly reduces depressive symptoms among both women and men, though the effect is stronger for women (–0.3794, *P*<.01) than for men (–0.2931, *P*<.05). Taken together, these findings highlight the differentiated impacts of the “Broadband China” policy, showing that middle-aged adults, less-educated individuals, and women derive relatively greater reductions in depressive symptoms, thereby underscoring the policy’s potential to mitigate health inequalities.

### Further Analysis

Table 8 presents the impact of digital infrastructure development on health inequality among older adults. Based on the Kakwani relative deprivation index, this study first reversely encodes the depressive symptoms variable, where higher scores indicate better health. Regression analysis is then conducted. Model 1 does not include control variables, while Models 2 and 3 introduce individual and family characteristics, respectively. Model 4 further considers time and regional fixed effects. In Model 4, the coefficient for the core explanatory variable did is –0.0089 (*P*<.01), indicating that digital infrastructure development significantly reduces inequalities in depressive symptoms among middle-aged and older adults. This finding confirms the role of digital infrastructure development in alleviating health inequality among middle-aged and older adults.

**Table 8:** The impact of digital infrastructure development on health inequality.

Variable		Model 1	Model 2	Model 3	Model 4
**Did**					
	β (95% CI)	–0.0119 (–0.0154 to –0.0083)	–0.0095 (–0.0130 to –0.0061)	–0.0103 (–0.0138 to –0.0068)	–0.0089 (–0.0140 to –0.0039)
	*P* value	<.001	<.001	<.001	.001
**Sex**					
	β (95% CI)	—^a^	–0.0513 (–0.0543 to –0.0483)	–0.0482 (–0.0512 to –0.0452)	–0.0488 (–0.0531 to –0.0446)
	*P* value	—	<.001	<.001	<.001
**Residence**					
	β (95% CI)	—	0.0240 (0.0209 to 0.0270)	0.0243 (0.0212 to 0.0273)	0.0242 (0.0187 to 0.0297)
	*P* value	—	<.001	<.001	<.001
**Age (years)**					
	β (95% CI)	—	0.0006 (0.0004 to 0.0008)	0.0001 (–0.0001 to 0.0003)	0.0003 (0.0000 to 0.0005)
	*P* value	—	<.001	.15	.03
**Education**					
	β (95% CI)	—	–0.0181 (–0.0196 to –0.0166)	–0.0179 (–0.0194 to –0.0164)	–0.0177 (–0.0198 to –0.0156)
	*P* value	—	<.001	<.001	<.001
**Ethnicity**					
	β (95% CI)	—	–0.0126 (–0.0193 to –0.0059)	–0.0120 (–0.0187 to –0.0054)	–0.0097 (–0.0235 to 0.0041)
	*P* value	—	<.001	<.001	.17
**Pension**					
	β (95% CI)	—	–0.0054 (–0.0088 to –0.0019)	–0.0050 (–0.0085 to –0.0016)	–0.0088 (–0.0125 to –0.0050)
	*P* value	—	.002	<.001	<.001
**Health insurance**					
	β (95% CI)	—	–0.0220 (–0.0292 to –0.0147)	–0.0190 (–0.0262 to –0.0118)	–0.0170 (–0.0258 to –0.0082)
	*P* value	—	<.001	<.001	<.001
**Chronic disease**					
	β (95% CI)	—	0.0590 (0.0557 to 0.0624)	0.0594 (0.0560 to 0.0628)	0.0557 (0.0519 to 0.0594)
	*P* value	—	<.001	<.001	<.001
**Marital status**					
	β (95% CI)	—	—	–0.0396 (–0.0443 to –0.0350)	–0.0397 (–0.0471 to –0.0323)
	*P* value	—	—	<.001	<.001
**Household size**					
	β (95% CI)	—	—	–0.0021 (–0.0030 to –0.0012)	–0.0020 (–0.0032 to –0.0009)
	*P* value	—	—	<.001	<.001
**_cons**					
	β (95% CI)	0.1429 (0.1413 to 0.1446)	0.1432 (0.1279 to 0.1585)	0.2080 (0.1911 to 0.2249)	0.2003 (0.1757 to 0.2248)
	*P* value	<.001	<.001	<.001	<.001
Time-fixed effects		No	No	No	Yes
Regional-fixed effects		No	No	No	Yes
*N*		56,211	56,211	56,211	56,211
Adjusted *R*^2^		0.0008	0.0750	0.0804	0.0946

^a^Not available.

## Discussion

### Principal Findings

This study uses panel data from CHARLS 2011-2020 and adopts the DID method, based on the “Broadband China” pilot policy, to assess the impact of digital infrastructure development on the depressive symptoms of middle-aged and older adults, analyze its mechanisms, and explore its effects on health inequality in this group. The findings are as follows:

First, digital infrastructure development has been shown to reduce depressive symptoms of middle-aged and older adults. The findings of this study remain robust even after controlling for selection bias and unobserved factors, providing further evidence of the association effect of digital infrastructure development on the depressive symptoms of older adults. This result is consistent with existing literature, which highlights that digital technologies can effectively alleviate depression [54], a conclusion that applies to various populations, such as children [55], adolescents [56], and older adults [24]. However, the innovation of this study lies in its departure from the traditional focus on individual or household-level perspectives, instead examining the impact of digital infrastructure development on depressive symptoms from a macro policy perspective. This approach reveals the positive role of government-driven policy interventions aimed at expanding digital technology access in enhancing the well-being of the older adult population, further emphasizing the critical role of policy orientation in improving health outcomes.

Second, digital infrastructure development helps to alleviate depressive symptoms among middle-aged and older adults by promoting the improvement of their social networks. This finding is consistent with social network theory, which highlights the critical role of social connections in individual psychological health [57]. Existing research has confirmed that social networks provide emotional support, reduce feelings of loneliness, and enhance social identity [58]. However, while many studies focus on the impact of social networks on psychological health, few explore how digital infrastructure development can promote the formation and enhancement of social networks at a macro level. This study fills this gap by revealing the role of digital infrastructure development in strengthening the social networks of middle-aged and older adults, thereby mitigating depressive symptoms. It further underscores the systematic effect of policy interventions on promoting psychological well-being.

Further analysis shows that improvements in depressive symptoms among middle-aged and older adults are primarily achieved through 3 social network pathways: enhancing family connection, close social interactions, and social participation. The strengthening of these pathways effectively reinforces the social support system, ultimately leading to reduced depressive symptoms. Strengthening family connections is likely due to the reduction in communication costs provided by digital technologies, enabling more frequent and intimate interactions between middle-aged and older adults and their children or relatives [59]. Regarding close social interactions, the proliferation of digital platforms, especially social media, provides a more convenient channel for older adults to stay in touch with friends and expand their social circles, thereby alleviating feelings of loneliness and enhancing social support [60]. Digital technologies have also significantly promoted the social participation of middle-aged and older adults, particularly in community activities, web-based learning, and public affairs, which not only increases their sense of social role but also strengthens their sense of belonging [33]. These findings indicate that digital infrastructure development not only represents an investment in material resources but also has a profound influence on social relationships, highlighting the critical role of digital policies in reducing depressive symptoms among middle-aged and older adults.

Third, the effects of digital infrastructure development on depressive symptoms vary significantly across different subgroups. Specifically, the positive association between digital infrastructure development and improved psychological well-being is more pronounced among women, middle-aged adults, and those with lower education levels. Consistent with existing research, the health improvements in these groups may stem from the promotion of social welfare and individual lifestyle changes facilitated by digital infrastructure development [61,62]. However, this study further reveals the underlying reasons why these groups benefit more.

The significant benefits observed in middle-aged adults suggest that the impact of digital infrastructure development on this group is closely related to the awakening of their health awareness and changes in behavior. Middle-aged adults are typically at a critical transitional stage in life, facing pressures related to health management and the dual burdens of work and family. Digital infrastructure can provide this group with more convenient access to health information and services, which helps mitigate depressive symptoms. Therefore, health interventions targeted at middle-aged adults should focus more on the application of digital tools to enhance health awareness and promote behavior change.

The significant benefits observed among individuals with lower education levels reveal the key role of education in digital technology acceptance and use. Lower-educated groups often face higher health risks, and the simple and user-friendly health tools and educational content provided by digital technologies can effectively reduce cognitive and technological barriers [63]. These groups’ ability to access health information and manage their health is enhanced through digital infrastructure. Therefore, digital health interventions targeted at lower-educated groups should focus more on designing health tools that align with their cognitive abilities and technical skills to ensure effective participation and benefit.

At the same time, this study also identifies significant sex differences. Digital infrastructure development has a positive impact on reducing depressive symptoms for both men and women, but the effect is more pronounced among women. This finding may be linked to the multiple responsibilities and pressures that women often bear in both family and social roles [64]. The improvement of digital infrastructure not only provides more opportunities for social interaction but also facilitates access to health resources and psychological support, thereby helping to alleviate women’s emotional distress and mental burden to some extent. Therefore, in advancing digital infrastructure development, greater attention should be directed toward addressing the specific needs of women, making use of digital tools to enhance their health and psychological support, and ultimately promoting sex equity in health outcomes.

In contrast, the effects on older adults and those with higher education levels are relatively weaker, which may be closely related to their existing health resources and methods of accessing information. Older adults often face multiple challenges with their health, but they may have higher barriers to adapting to new technologies [65]. Therefore, although digital infrastructure can provide convenience for older adults, issues such as technological adaptability and the learning curve may affect their health benefits. Additionally, urban residents and those with higher education levels are generally more easily able to access medical services and health information, meaning the marginal benefits of digital infrastructure development are lower in these groups.

Fourth, digital infrastructure development has alleviated health inequality in depressive symptoms among middle-aged and older adults to some extent. Previous studies have shown that health inequality is influenced by various factors, including education level [66] and geographic location [67]. However, most existing literature focuses on how traditional socioeconomic factors exacerbate health inequality, with less emphasis on how digital infrastructure can bridge gaps among different social groups by optimizing the allocation of public resources and improving the accessibility of services related to depression. This study finds that digital infrastructure development has had a significant positive effect on disadvantaged groups, demonstrating a certain “compensatory effect.” A possible explanation is that traditional health services are often limited by spatial, temporal, and financial constraints, whereas digital infrastructure can provide remote medical care, health education, and information access, significantly reducing the geographical and economic barriers faced by disadvantaged groups and improving their access to health services. Moreover, the ease of use of digital technologies and the convenience of information delivery help reduce the cognitive burden faced by groups with lower health literacy during health decision-making, thereby enhancing their ability to manage depressive symptoms independently. This “compensatory effect” suggests that under the promotion of digital infrastructure development, traditionally disadvantaged groups can better access health services and improve their health outcomes. This not only helps reduce health disparities among groups but also demonstrates the enormous potential of digital technology in promoting health equity.

This study has several strengths. First, it innovatively identifies the associations between digital technology and depressive symptoms. Based on the natural experiment of the “Broadband China” pilot policy, the study uses the DID method to address endogeneity issues and provides external validity evidence of the effects of digital infrastructure development on the depressive symptoms of middle-aged and older adults, filling the gap in existing research regarding the impact of macro digital policy interventions on depression. Second, it explores the social network mechanism in depth. The study reveals how digital infrastructure promotes family connections, close social interactions, and social participation to mitigate depressive symptoms among middle-aged and older adults, enriching the theoretical framework of the interaction between digital infrastructure and social networks. It provides empirical evidence for policymakers to improve the well-being of middle-aged and older adults while promoting social network development. Third, it expands the perspective of health inequality. By measuring disparities in depressive symptoms and conducting heterogeneity analysis from multiple perspectives, the study explores the “compensatory effect” of digital infrastructure development on low-resource groups, providing strong empirical support for healthy aging policies and emphasizing the tremendous potential of digital technology in reducing health disparities.

However, the study has some limitations. First, the data used in this study were primarily collected and assessed through self-reports or proxy reports, which may introduce recall bias and subjectivity, especially concerning depressive symptoms and social network-related issues. Nevertheless, the CHARLS survey follows standardized protocols, ensuring the reliability and comparability of cross-wave data, thus effectively reducing potential biases. Second, due to limitations in data updates, this study was unable to obtain the CHARLS database after 2020. Therefore, future research could extend the time frame and further explore the long-term effects of digital infrastructure on depressive symptoms among middle-aged and older adults. Third, this study did not analyze other potential factors that may affect depression, such as the expansion of long-term care pilot projects and the improvement of health literacy. These factors may influence depressive symptoms to some extent. Future research should incorporate more diversified data sources to further assess the associations between these factors and depression among middle-aged and older adults.

### Conclusions

This study, based on panel data from CHARLS 2011-2020, uses a quasi-natural experiment based on the “Broadband China” pilot policy to evaluate the impact of digital infrastructure development on the depressive symptoms of middle-aged and older adults. It also explores the role of social networks and subgroup heterogeneity. The key conclusions are as follows:

First, digital infrastructure development significantly improves the depressive symptoms of middle-aged and older adults, confirming the effectiveness of digital technology in reducing depression. The study analyzes the positive role of government-driven digital infrastructure development from a macro policy perspective, emphasizing the critical role of policy guidance in promoting reductions in depressive symptoms and improving psychological well-being. Second, digital infrastructure development alleviates depressive symptoms by strengthening the social networks of middle-aged and older adults. The study finds that digital infrastructure effectively promotes family connection, close social interactions, and social participation, thereby enhancing social support systems. This indicates that digital infrastructure not only impacts material aspects but also has profound effects at the social relationship level. Third, the impact of digital infrastructure development on depressive symptoms across different subgroups exhibits significant heterogeneity, particularly for women, middle-aged adults, and low-education groups. This suggests that digital infrastructure development helps these groups overcome barriers to health information access and health management, thereby reducing depression levels. Fourth, digital infrastructure development plays an important role in alleviating health inequality. By increasing the accessibility of health services, it significantly reduces depressive symptoms among low-resource groups, thereby improving their psychological well-being, demonstrating the “compensatory effect” of digital infrastructure.

Based on these conclusions, the study proposes the following policy recommendations. First, digital infrastructure should be embedded in long-term national strategies, with coordinated governance and financial investment to reduce regional disparities. Such efforts resonate with the World Health Organization’s Global Strategy on Digital Health and highlight the importance of aligning national initiatives with global frameworks. Second, improving digital literacy across the life course is equally crucial. Lifelong, tailored training—especially for women, middle-aged adults, and low-education groups—should combine web-based platforms with community-based programs, drawing on international experiences such as the EU’s Active and Healthy Ageing initiatives. Third, strengthening social networks through digital platforms can further alleviate depressive symptoms. Public-private partnerships that encourage family interaction, community participation, and intergenerational support have proven effective in other contexts, such as Japan and Europe, and can be adapted locally. Finally, digital health services should be fully integrated into primary care systems. Expanding reimbursement for telemedicine, embedding digital depression-related services into community health centers, and prioritizing underserved regions, as demonstrated in Australia’s telehealth reforms, can promote inclusivity and reduce health inequalities.

## Appendix

Multimedia Appendix 1 Descriptive statistics by survey wave.

## Abbreviations

CES-D: Center for Epidemiologic Studies Depression Scale
CHARLS: China Health and Retirement Longitudinal Study
DID: difference-in-differences
PSM-DID: propensity score matching difference-in-differences
VIF: variance inflation factors

## Data Availability

The data used in this study are available from the China Health and Retirement Longitudinal Study (CHARLS) repository [68]. Researchers may request access following the procedures described on the official website.

## References

[1] Partridge L, Deelen J, Slagboom PE (2018). Facing up to the global challenges of ageing. Nature.

[2] Webb LM, Chen CY (2022). The COVID-19 pandemic's impact on older adults' mental health: contributing factors, coping strategies, and opportunities for improvement. Int J Geriatr Psychiatry.

[3] Abdoli N, Salari N, Darvishi N, Jafarpour S, Solaymani M, Mohammadi M, Shohaimi S (2022). The global prevalence of major depressive disorder (MDD) among the elderly: a systematic review and meta-analysis. Neurosci Biobehav Rev.

[4] Fan X, Guo X, Ren Z, Li X, He M, Shi H, Zha S, Qiao S, Zhao H, Li Y, Pu Y, Liu H, Zhang X (2021). The prevalence of depressive symptoms and associated factors in middle-aged and elderly Chinese people. J Affect Disord.

[5] Wang F, Zhang Q, Zhang L, Ng CH, Ungvari GS, Yuan Z, Zhang J, Zhang L, Xiang Y (2018). Prevalence of major depressive disorder in older adults in China: a systematic review and meta-analysis. J Affect Disord.

[6] Proudman D, Greenberg P, Nellesen D (2021). The growing burden of major depressive disorders (MDD): implications for researchers and policy makers. Pharmacoeconomics.

[7] Arias D, Saxena S, Verguet S (2022). Quantifying the global burden of mental disorders and their economic value. EClinicalMedicine.

[8] Vigo D, Jones L, Atun R, Thornicroft G (2022). The true global disease burden of mental illness: still elusive. Lancet Psychiatry.

[9] Banerjee A, Duflo E, Grela E, McKelway M, Schilbach F, Sharma G, Vaidyanathan G (2023). Depression and loneliness among the elderly in low- and middle-income countries. J Econ Perspect.

[10] Marinucci M, Pancani L, Aureli N, Riva P (2022). Online social connections as surrogates of face-to-face interactions: a longitudinal study under Covid-19 isolation. Comput Hum Behav.

[11] Li Z (2024). Analysis of the health effects of multiple social networks on the older adult: the substitution role of labor participation. Front Public Health.

[12] Boardman J, Killaspy H, Mezey G (2022). Social Inclusion and Mental Health.

[13] Li C, Ning G, Xia Y, Guo K, Liu Q (2022). Does the internet bring people closer together or further apart? the impact of internet usage on interpersonal communications. Behav Sci (Basel).

[14] Zhang K, Burr J, Mutchler J, Lu J (2024). Internet use and loneliness among urban and non-urban Chinese older adults: the roles of family support, friend support, and social participation. J Gerontol B Psychol Sci Soc Sci.

[15] Chai Y, Xian G, Wang M, Guo L, Luo S (2024). Aging wisely: the impact of internet use on older adults' mental health. J Affect Disord.

[16] Litwin H, Levinsky M (2022). Social networks and mental health change in older adults after the Covid-19 outbreak. Aging Ment Health.

[17] Hardy BW, Castonguay J (2018). The moderating role of age in the relationship between social media use and mental well-being: an analysis of the 2016 general social survey. Comput Hum Behav.

[18] Li X, He P, Liao H, Liu J, Chen L (2024). Does network infrastructure construction reduce urban–rural income inequality? based on the “Broadband China” policy. Technol Forecast Soc Change.

[19] Xu Q, Li X, Dong Y, Guo F (2025). How digital infrastructure development affects residents' health: a quasi-natural experiment based on the “Broadband China” strategy. Cities.

[20] Zhou Y, Bai Y, Wang J (2024). The impact of internet use on health among older adults in China: a nationally representative study. BMC Public Health.

[21] Ouden WV, van Boekel L, Janssen M, Leenders R, Luijkx K (2021). The impact of social network change and health decline: a qualitative study on experiences of older adults who are ageing in place. BMC Geriatr.

[22] Lapshin I (2018). Digital resocialization of elderly people. Proceedings of the International Conference on Contemporary Education, Social Sciences and Ecological Studies (CESSES 2018).

[23] Tsang APL, Lee CK, Chan SCY (2025). A cross-lagged panel analysis of social participation in the relationship between functional limitations and cognitive functioning: evidence from CHARLS. J Appl Gerontol.

[24] Anderer S (2025). Internet use may boost mental health benefits in older adults. JAMA.

[25] Yang H, Zhang S, Cheng S, Li Z, Wu Y, Zhang S, Wang J, Tao Y, Yao Y, Xie L, Xiao W, Tang X, Wu J, Shen Z, Tang L (2022). A study on the impact of internet use on depression among Chinese older people under the perspective of social participation. BMC Geriatr.

[26] Drageset J (2021). Social support. Health Promotion in Health Care – Vital Theories and Research.

[27] Hajek A, König HH (2022). Frequency of contact with friends and relatives via internet and psychosocial factors in middle-aged and older adults during the COVID-19 pandemic. findings from the German ageing survey. Int J Geriatr Psychiatry.

[28] Wrzus C, Hänel M, Wagner J, Neyer FJ (2013). Social network changes and life events across the life span: a meta-analysis. Psychol Bull.

[29] Kennedy Terry KM (2022). At the intersection of SLA and sociolinguistics: the predictive power of social networks during study abroad. Mod Lang J.

[30] Granovetter MS (1973). The strength of weak ties. Am J Sociol.

[31] Jaspal R, Breakwell GM (2022). Socio-economic inequalities in social network, loneliness and mental health during the COVID-19 pandemic. Int J Soc Psychiatry.

[32] Wu Z, Penning MJ (2018). Children and the mental health of older adults in China: what matters?. Popul Res Policy Rev.

[33] Sun K, Zhou J (2021). Understanding the impacts of internet use on senior citizens’ social participation in China: evidence from longitudinal panel data. Telemat Inform.

[34] Peng Z, Dan T (2023). Digital dividend or digital divide? digital economy and urban-rural income inequality in China. Telecommun Policy.

[35] Francis DV, Weller CE (2022). Economic inequality, the digital divide, and remote learning during COVID-19. Rev Black Polit Econ.

[36] van de Werfhorst HG, Kessenich E, Geven S (2022). The digital divide in online education: inequality in digital readiness of students and schools. Comput Educ Open.

[37] Cheshmehzangi A, Zou T, Su Z (2022). The digital divide impacts on mental health during the COVID-19 pandemic. Brain Behav Immun.

[38] Li H (2024). Asymmetric information in the field of healthcare. Adv Econ Manag Political Sci.

[39] Sundvall M, Titelman D, DeMarinis V, Borisova L, Çetrez Ö (2021). Safe but isolated - an interview study with Iraqi refugees in Sweden about social networks, social support, and mental health. Int J Soc Psychiatry.

[40] Chen E, Wood D, Ysseldyk R (2022). Online social networking and mental health among older adults: a scoping review. Can J Aging.

[41] Li Z, Zheng L, Xu H (2025). From caregiving burden to income transformation: intergenerational employment effects of long-term care insurance. Front Public Health.

[42] Chu J, Li Y, Wang X, Xu Q, Xu Z (2025). Development of a longitudinal model for disability prediction in older adults in China: analysis of CHARLS data (2015-2020). JMIR Aging.

[43] Peng H, Ling K, Zhang Y (2024). The carbon emission reduction effect of digital infrastructure development: evidence from the broadband China policy. J Clean Prod.

[44] He J, Mu Y, Wang C, Mao Y (2024). Impact of digital infrastructure construction on financial development: evidence from the "broadband China" strategy. Heliyon.

[45] Jia X, Li X (2024). Has digital infrastructure accelerated enterprises' specialization? evidence from China. Bus Process Manag J.

[46] Ma N, Ji X, Shi Y, Wang Q, Wu J, Cui X, Niu W (2024). Adverse childhood experiences and mental health disorder in China: a nationwide study from CHARLS. J Affect Disord.

[47] Zhang X, Xue M, Zhang Z, Gao Z, Li C, Wu J, Niu W (2024). Impact of social, familial and personal factors on depressive symptoms in middle-aged and older adults from the national CHARLS cohort. BMC Public Health.

[48] Chen H, Mui AC (2014). Factorial validity of the center for epidemiologic studies depression scale short form in older population in China. Int Psychogeriatr.

[49] Beck T, Levine R, Levkov A (2010). Big bad banks? the winners and losers from bank deregulation in the United States. J Finance.

[50] Kakwani N, Wagstaff A, van Doorslaer E (1997). Socioeconomic inequalities in health: measurement, computation, and statistical inference. J Econom.

[51] Turguttopbaş N (2020). Analysis of the equity of health care financing system in Turkey. Turk Stud.

[52] Jeong HJ, Kim S, Lee J (2021). Mental health, life satisfaction, supportive parent communication, and help-seeking sources in the wake of COVID-19: first-generation college students vs. non-first-generation college students. J Coll Stud Psychother.

[53] Headey B, Kelley J, Wearing A (1993). Dimensions of mental health: life satisfaction, positive affect, anxiety and depression. Soc Indic Res.

[54] Li J (2023). Digital technologies for mental health improvements in the COVID-19 pandemic: a scoping review. BMC Public Health.

[55] Cardoso-Leite P, Buchard A, Tissieres I, Mussack D, Bavelier D (2021). Media use, attention, mental health and academic performance among 8 to 12 year old children. PLoS One.

[56] Metherell TE, Ghai S, McCormick EM, Ford TJ, Orben A (2022). Digital access constraints predict worse mental health among adolescents during COVID-19. Sci Rep.

[57] Wellman B (1979). The community question: the intimate networks of east yorkers. Am J Sociol.

[58] Kim H, Kwak S, Youm Y, Chey J (2022). Social network characteristics predict loneliness in older adults. Gerontology.

[59] Lu H, Kandilov IT (2021). Does mobile internet use affect the subjective well-being of older chinese adults? an instrumental variable quantile analysis. J Happiness Stud.

[60] Yu B, Leung YW (2023). Establishing ties or strengthening friendships? students' use of online social networks in intercultural friendship development. Inf Technol People.

[61] Peláez AL, Suh SM, Zelenev S (2022). Digital Transformation and Social Well-Being: Promoting an Inclusive Society.

[62] Hatuka T, Zur H, Mendoza JA (2021). The urban digital lifestyle: an analytical framework for placing digital practices in a spatial context and for developing applicable policy. Cities.

[63] Weßner A, Mohajerzad H, Fliegener L, Bernhard-Skala C, Rohs M (2024). Consensus in uncertainty. a group Delphi study on the impact of digitalisation on the continuing education of low-qualified adults in Germany. Int J Lifelong Educ.

[64] Mussida C, Patimo R (2021). Women's family care responsibilities, employment and health: a tale of two countries. J Fam Econ Issues.

[65] Tomczyk L, Mascia ML, Gierszewski D, Walker C (2023). Barriers to digital inclusion among older people: a intergenerational reflection on the need to develop digital competences for the group with the highest level of digital exclusion. Int J Technol Educ Innov.

[66] Popham F, Iannelli C (2021). Does comprehensive education reduce health inequalities?. SSM Popul Health.

[67] Akter M, Kabir H (2023). Health inequalities in rural and urban Bangladesh: the implications of digital health. Mayo Clin Proc Digit Health.

[68] China Health and Retirement Longitudinal Study.

