# Supplementary material for: Digital Pathways to Reducing Depression Among Aging Populations Through the “Broadband China” Pilot Program: Quasi-Natural Experiment
Source: JMIR Aging. 2025 Oct 27;8:e79260. doi: 10.2196/79260 (PMC12603584; doi:10.2196/79260)
Supplement: Multimedia Appendix 1 [file aging_v8i1e79260_app1.docx]

Table S1. Descriptive Statistics by Survey Wave

| Year | Variable Name | Mean | SD | Min | Max |
| --- | --- | --- | --- | --- | --- |
| 2011 | depressive symptoms | 7.933 | 6.053 | 0 | 30 |
|  | did | 0 | 0.000 | 0 | 0 |
|  | residence | .635 | 0.481 | 0 | 1 |
|  | gender | .503 | 0.500 | 0 | 1 |
|  | age | 58.432 | 8.426 | 46 | 101 |
|  | education | 2.048 | 1.084 | 1 | 4 |
|  | ethnicity | .953 | 0.211 | 0 | 1 |
|  | pension | .326 | 0.469 | 0 | 1 |
|  | health insurance | .95 | 0.217 | 0 | 1 |
|  | chronic disease | .657 | 0.475 | 0 | 1 |
|  | marital status | .91 | 0.287 | 0 | 1 |
|  | household size | 3.606 | 1.805 | 1 | 16 |
| Year | Variable Name | Mean | SD | Min | Max |
| 2013 | depressive symptoms | 7.672 | 5.601 | 0 | 30 |
|  | did | 0 | 0.000 | 0 | 0 |
|  | residence | .64 | 0.480 | 0 | 1 |
|  | gender | .49 | 0.500 | 0 | 1 |
|  | age | 60.171 | 8.983 | 46 | 103 |
|  | education | 2.01 | 1.067 | 1 | 4 |
|  | ethnicity | .954 | 0.210 | 0 | 1 |
|  | pension | .495 | 0.500 | 0 | 1 |
|  | health insurance | .971 | 0.168 | 0 | 1 |
|  | chronic disease | .705 | 0.456 | 0 | 1 |
|  | marital status | .891 | 0.312 | 0 | 1 |
|  | household size | 3.62 | 1.808 | 1 | 15 |
| Year | Variable Name | Mean | SD | Min | Max |
| 2015 | depressive symptoms | 7.86 | 6.221 | 0 | 30 |
|  | did | .259 | 0.438 | 0 | 1 |
|  | residence | .656 | 0.475 | 0 | 1 |
|  | gender | .5 | 0.500 | 0 | 1 |
|  | age | 61.468 | 8.958 | 46 | 105 |
|  | education | 2.009 | 1.054 | 1 | 4 |
|  | ethnicity | .952 | 0.214 | 0 | 1 |
|  | pension | .518 | 0.500 | 0 | 1 |
|  | health insurance | .936 | 0.245 | 0 | 1 |
|  | chronic disease | .794 | 0.405 | 0 | 1 |
|  | marital status | .879 | 0.326 | 0 | 1 |
|  | household size | 3.037 | 1.348 | 1 | 15 |
| Year | Variable Name | Mean | SD | Min | Max |
| 2018 | depressive symptoms | 8.12 | 6.205 | 0 | 30 |
|  | did | .357 | 0.479 | 0 | 1 |
|  | residence | .605 | 0.489 | 0 | 1 |
|  | gender | .499 | 0.500 | 0 | 1 |
|  | age | 62.098 | 9.431 | 46 | 108 |
|  | education | 2.061 | 1.032 | 1 | 4 |
|  | ethnicity | .951 | 0.216 | 0 | 1 |
|  | pension | .62 | 0.486 | 0 | 1 |
|  | health insurance | .976 | 0.154 | 0 | 1 |
|  | chronic disease | .801 | 0.399 | 0 | 1 |
|  | marital status | .868 | 0.339 | 0 | 1 |
|  | household size | 2.777 | 1.439 | 1 | 13 |
| Year | Variable Name | Mean | SD | Min | Max |
| 2020 | depressive symptoms | 8.511 | 6.314 | 0 | 30 |
|  | did | .358 | 0.479 | 0 | 1 |
|  | residence | .602 | 0.489 | 0 | 1 |
|  | gender | .482 | 0.500 | 0 | 1 |
|  | age | 62.701 | 9.199 | 46 | 120 |
|  | education | 2.119 | 1.073 | 1 | 4 |
|  | ethnicity | .951 | 0.216 | 0 | 1 |
|  | pension | .858 | 0.349 | 0 | 1 |
|  | health insurance | .958 | 0.201 | 0 | 1 |
|  | chronic disease | .806 | 0.396 | 0 | 1 |
|  | marital status | .868 | 0.339 | 0 | 1 |
|  | household size | 2.962 | 1.582 | 1 | 16 |
